# Evidence of ectopic recombination and a repeat-induced point (RIP) mutation in the genome of *Sclerotinia sclerotiorum*, the agent responsible for white mold

**DOI:** 10.1590/1678-4685-GMB-2015-0241

**Published:** 2016-07-07

**Authors:** Míriam Goldfarb, Mateus Ferreira Santana, Tânia Maria Fernandes Salomão, Marisa Vieira de Queiroz, Everaldo Gonçalves de Barros

**Affiliations:** 1 Laboratório de Biologia Molecular de Plantas, Instituto de Biotecnologia Aplicada (BIOAGRO), Universidade Federal de Viçosa (UFV), 36570-000, Viçosa, MG, Brazil; 2 Laboratório de Genética Molecular e de Microrganismo, Instituto de Biotecnologia Aplicada (BIOAGRO), Universidade Federal de Viçosa (UFV), 36570-000, Viçosa, MG, Brazil; 3 Laboratório de Biologia Molecular de Insetos, Departamento de Biologia Geral, Universidade Federal de Viçosa (UFV), 36570-000, Viçosa, MG, Brazil; 4 Laboratório de Genética Molecular e de Microrganismo, Instituto de Biotecnologia Aplicada (BIOAGRO), Universidade Federal de Viçosa (UFV), 36570-000, Viçosa, MG, Brazil; 5 Laboratório de Ciências Genômicas e Biotecnologia, Universidade Católica de Brasília, 70790-160, Brasília, DF, Brazil

**Keywords:** phytopathogens, retrotransposons, transposable elements

## Abstract

Two retrotransposons from the superfamilies *Copia* and *Gypsy* named as *Copia-LTR_SS* and *Gypsy*-*LTR_SS*, respectively, were identified in the genomic bank of *Sclerotinia sclerotiorum*. These transposable elements (TEs) contained direct and preserved long terminal repeats (LTR). Domains related to codified regions for gag protein, integrase, reverse transcriptase and RNAse H were identified in *Copia-LTR_SS*, whereas in *Gypsy*-*LTR_SS* only domains for gag, reverse transcriptase and RNAse H were found. The abundance of identified LTR-Solo suggested possible genetic recombination events in the *S. sclerotiorum* genome. Furthermore, alignment of the sequences for LTR elements from each superfamily suggested the presence of a RIP (repeat-induced point mutation) silencing mechanism that may directly affect the evolution of this species.

Transposable elements (TEs) are ubiquitous DNA sequences in the genome that have the ability to move from one place to another (Kidwell, 2005). TEs form two classes based on the transposition mechanisms involved: class I includes the TEs usually referred to as retrotransposons and class II contains the "DNA transposons" *per se*. All class I TEs are transposed by intermediate RNA which is transcribed from a copy of the genome and the cDNA is obtained from a reverse transcriptase codified by the element itself. Every complete transposon cycle produces a new copy and, consequently, retrotransposons are frequently the main contributors to a repetitive fraction of the genome. Retrotransposons can be classified in five groups based on their mechanism of transposition and on the organization and phylogeny of the reverse transcriptase: LTR (long terminal repeat), DIRS-like (*Dictyostelium* intermediate repeat sequence), Penelope-like, LINEs (long interspersed nuclear elements) and SINEs (short interspersed nuclear elements) (Wicker *et al.*, 2007).

LTR retrotransposons are usually found in fungi, especially in the superfamilies *Gypsy* and *Copia*. The LTR *gag* and *pol* regions are structural compounds of the *Copia* and *Gypsy* retrotransposons. The LTRs flank the 5' and 3' extremities that are identical with active retrotransposons. The *gag* region encodes structural proteins similar to those of the viral capsid. The *pol* region encodes a polyprotein that is processed to yield the proteins involved in the transposition. These proteins include a protease involved in protein maturation and cleavage, a reverse transcriptase that reverse-transcribes the RNA into cDNA, an integrase that allows transposon insertion into the genome, and an RNAse H that degrades the RNA regions during cDNA synthesis. In addition, the PPT (polypurine tract) and PBS (primer binding site) regions facilitate transposon transcription in the genus (Havecker *et al.*, 2004; Manetti *et al.*, 2007). The *Gypsy* and *Copia* retrotransposons differ from each other in the arrangement of the sequence that encodes the reverse transcriptase and integrase (Wicker *et al.*, 2007).

TEs activities in the genome may affect gene structure and its regulation (Shapirova, 2010; Huan-Van *et al.*, 2011). In addition, TEs provide important sites for ectopic recombination in the genome (Dean *et al.*, 2005; Ohm *et al.*, 2012). In this regard, the genomes of organisms have different strategies to avoid possible damage caused by TEs present in the genome, including a silencing mechanism known as RIP (repeat-induced point mutation) that was originally discovered in *Neurospora crassa* (Selker, 1990, 2002). RIP occurs during the sexual cycle, between fertilization and karyogamy, and induces GC-to-AT mutations in duplicated DNA sequences longer than 400 pb and with an identity of > 80% (Galagan and Selker, 2004).

In most fungal species, TEs generally represent 2-20% of the genome, although in some cases they can account for 85% of the genome (Parlange *et al.*, 2011). Transposons are important elements for evolution of the genome in phytopathogenic fungi because of their linked gain or loss of virulence (Khang *et al.*, 2008; Chuma *et al.*, 2011). Many effector genes in plant pathogens occur in genomic regions that are rich in TEs. These ETs may alter the gene structure or expression and stimulate the emergence of new pathogenic races (Bakkeren and Valent, 2014). In addition, the presence of cognate-TEs in conserved domains of genes can lead to their integration into regulatory reticulations via microRNA (Li *et al.*, 2011).

*Sclerotinia sclerotiorum*, the causal agent of white mold, has a worldwide distribution with a range of hosts that consists of at least 408 species and 278 plant genera. Analyses of the genetic diversity of *S. sclerotiorum* TEs have suggested recent genomic remodeling involving TE expansion (Amselem *et al.*, 2011; Santana *et al.*, 2014a). The *S. sclerotiorum* genome is estimated to contain 38 Mb, 7% of which consists of TEs, with the frequency of LTR-retrotransposons being ~2-2.5% (Amselem *et al.*, 2011). In this work, we investigated the possible evolutionary impacts of TEs in the *S. sclerotiorum* genome.

The genomic sequences of *S. sclerotiorum* class I transposable elements were obtained by searching the fungal genome database (http://www.ncbi.nlm.nih.gov/assembly/GCF_000146945.1/) and using the LTR-Finder software. Subsequently, the remaining copies of the elements were obtained by using the Basic Local Alignment Search Tool (BLAST) for each previously identified element against the *S. sclerotiorum* genome. The main domains related to TE-encoded regions were tagged with the BLASTX tool (http://www.ncbi.nlm.nih.gov). TEs were classified based on their structural features and by phylogenetic sequence analysis that encoded the reverse transcriptase protein. The neighbor-joining method with a bootstrap value of 5,000 replications was used for the phylogenetic analysis and included the reverse transcriptase protein sequence from different TE groups: *Maggy* from *Magnaporthe grisea* (AAA33420), *Real* from *Alternaria alternata* (BAA89272), *Ty3* from *Saccharomyces cerevisiae* (M23367), *copia* from *Drosophila simulans* (D10880), *Ty1* from *S. cerevisiae* (Z48149), *jockey* from *Drosophila melanogaster* (M22874), *Penelope* from *Drosophila virilis* (AAL14979) and *DIRS* from *Lytechinus variegatus* (BK001257). The sequence alignment and phylogenetic analysis were done using MEGA4 software (Tamura *et al.*, 2007).

Evidence for a RIP silencing mechanism was obtained from an analysis of 157 sequences from *Copia-LTR_SS* retrotransposons and 12 sequences from *Gypsy*-*LTR_SS* retrotransposon*s.* The sequences were aligned using MEGA4 software (Tamura *et al.*, 2007). The dinucleotide frequency analysis and estimation of the RIP indices were determined using RipCal software (Hane and Oliver, 2008). The indices or ratios used to prove RIP were TpA/ApT and (CpA+TpG)/(ApC+GpT). The data obtained for *S. sclerotiorum* were compared to the transposase sequences of *Colletrotrichum cereale* (Crouch *et al.*, 2008), the PeTra element of *Penicillium chrysogenum* (Braumann *et al.*, 2008), element OPUIO3-1414 of *Ophiostoma novo-ulmi* (Bouvet *et al.*, 2008), element *Fot 1* of *Fusarium oxysporum* (Daboussi *et al.*, 1992) and element *Punt* of *N. crassa* (Magolin *et al.*, 1998) by using the same indices.

Examination of the *S. sclerotiorum* genome revealed two retrotransposons possibly involved in the restructuring of the fungal genome. The structural and phylogenetic analyses (Figure 1) of these two elements allowed their classification as part of the superfamilies *Copia* and *Gypsy*; the elements were referred to as *Copia-LTR_SS* (supercontig 8: 23.5003-24.0346) and *Gypsy-LTR_SS* (supercontig 36: 1.308-7.775) (Figure S1, Supplementary material). A total of seven complete elements were identified, six of which belonged to the *Copia* element and one to the *Gypsy* element. The *Copia-LTR_SS* element (5,344 bp) ` long terminal repetitions (LTRs) that were directly conserved (269 bp) and conserved domains that encoded *gag* and *pol* region proteins such as integrase, reverse transcriptase and RNAse H (Figure 2A).

**Figure 1:**
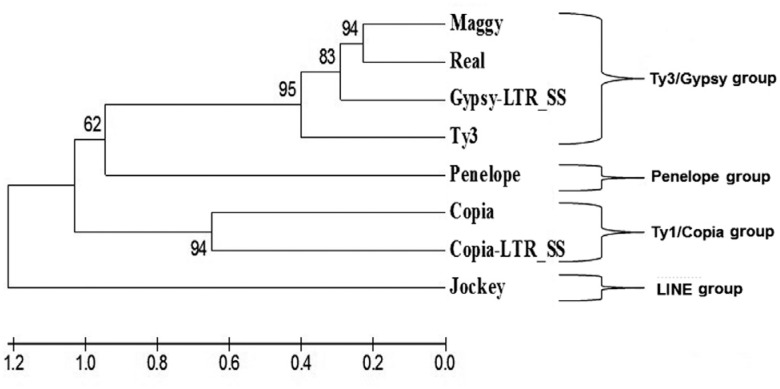
A dendrogram showing the grouping of the *Copia-LTR_SS* and *Gypsy*-*LTR_SS* elements. The analysis was done using the neighbor-joining method based on 5,000 bootstrap replicates. The numbers above and below each node indicate the percentage of times in which each branch appeared in a bootstrap analysis with 5,000 replicates. X-axis numbers refer to genetic divergence.

**Figure 2:**
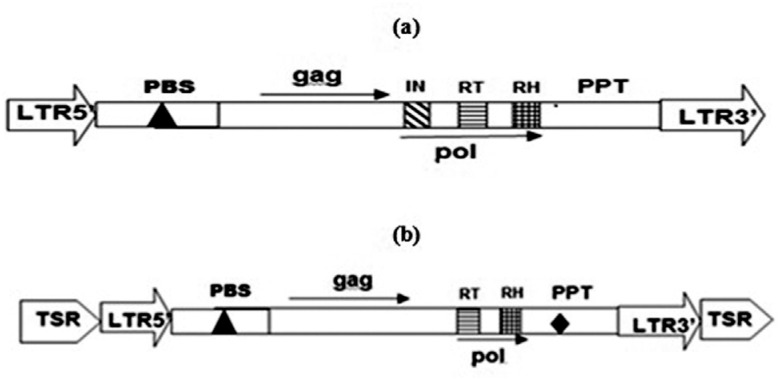
Structural organization of the retrotransposons *Copia-LTR_SS* (**A**) and *Gypsy-LTR_SS* (**B**) identified in the genome of *S. sclerotiorum*. The *pol* region of *Copia-LTR_SS* contained domains for integrase (IN), reverse transcriptase (RT) and RNAse H (RH), whereas *Gypsy-LTR_SS* had only RT and RH domains. The two elements had PBS (primer binding site) and PPT (polypurine tract) regions. Large arrows represent the LTRs.

The type of protein and its position in the open-reading frame (ORF) were typical of elements from the superfamily *Copia.* The conserved LTRs (435 bp) and the conserved domain for the *Gag* protein, the reverse transcriptase and the RNAse H were also found in the *Gypsy*-*LTR_SS* element (6,468 bp). Nonetheless, the domain containing the integrase and protease were not labeled in this element (Figure 2B). Mutations in this element are a possible explanation for the absence of the integrase and protease domains and will result in an inactive element. In addition, most of the retrotransposon sequences found in the *S. sclerotiorum* genome are degenerate (Amselem *et al.*, 2011). The conserved *Gypsy-LTR_SS* contained LTR 5' and 3' flanking insertion signs known as TSRs (target site repeats) that consisted of five base-pairs (GAAAT). These TSRs are duplicated TE target sequences that arise at the moment of insertion. In both elements, purine-rich regions known as PPT and PBS were identified. These regions are important for the reverse transposons of TE. An analysis of approximately 5,000 bp in the upstream and downstream sequences of the complete TEs demonstrated that the elements occurred in regions rich in repetitive sequences and were neighbors to genes related to mRNA splicing, apoptosis and heterokaryon incompatibility.

In all, 141 and 359 solo-LTR sequences were identified for the *Copia-LTR_SS* and *Gypsy-LTR_SS* retrotransposons, respectively. The presence of non-autonomous elements and solo-LTRs in *S. sclerotiorum* highlighted the possible occurrence of ectopic recombination in this fungal genome. The reason for this is that these sequences generally result in recombination between TE sequences and those of the same family. Additional evidence for recombination in the *S. sclerotiorum* genome involving TEs was the fact that the different TSRs flanked copies of the six identified C*opia-LTR_SS* elements. The presence of different insertion areas in the extremities of a single TE may reflect the recombination of similar retrotransposons containing different TSRs. Ectopic recombination through transposons has been reported as an important genome reconstruction event in many fungi, such as *Magnaporthe grisea* (Dean *et al.*, 2005), *Coprinopsis cinerea* (Stajich *et al.*, 2010), *Verticilium dahliae* (Amyotte *et al.*, 2012), *Mycosphaerella fijiensis* (Santana *et al.*, 2012) and *Cochliobolus heterostrophus* (Santana *et al.*, 2014b), among others.

TRIM (terminal-repeat retrotransposon in miniature) elements were also tagged: three sequences originated from the *Copia-LTR_SS* element and 21 from the *Gypsy*-*LTR_SS* element. These transposons result from autonomous LTR retroelements. However, the DNA sequences related to *pol* or *gag* region proteins are absent, making these elements defective (non-autonomous). However, these elements can move through the genome using the enzymatic machinery of similar elements (Wicker *et al.*, 2007).

The results of RIP analysis of the LTR sequences of *Copia-LTR_SS* and *Gypsy-LTR_SS* TEs were compared to those reported in the literature and their corresponding RIP mechanism (Table 1). The ratios TpA/ApT and (CpA + TpG)/(ApC+GpT) obtained for *S. sclerotiorum* were the same as those already reported in the literature. This finding suggested that the existing CpA-dinucleotides in the LTRs of suitable elements in *S. sclerotiorum* are the target of mutations generated by a process similar to the RIP mechanism. Evidence of RIP in *S. sclerotiorum* has also been provided by Amselem *et al.* (2015). In contrast, Santana *et al.* (2014a) found no RIP silencing mechanism in *Tc1-Mariner* elements (class II transposons) of the *S. sclerotiorum* genome. Together, these findings indicate variation in the occurrence of RIP silencing mechanisms among TEs in *S. sclerotiorum*. Similar behavior has been reported for the genomes of *Aspergillus niger* (Braumann *et al.*, 2008) and *C. heterostrophus* (Santana *et al.*, 2014b).

**Table 1:** TpA/ApT and CpA+TpG/ApC+GpT ratios for transposons and retrotransposons.

Sequences analyzed	(TpA/ApT)	(CpA+TpG)/(ApC+GpT)	Reference
Retrotransposon *Copia-LTR_SS*	1.35	0.27	This study
Retrotransposon Gypsy-*LTR_SS*	1.0	0.95	This study
Transposase (*Colletrotrichum cereale*)	2.00	0.44	Crouch *et al.* (2008)
*PetTra* (*Penicillium chrysogenum)*	1.22	0.58	Braumann *et al.* (2008)
OPHIO3-1414 (*Ophiostoma novo-ulmi*)	1.51	0.60	Bouvet *et al.* (2008)
Fot1 (*Fusarium oxysporum)*	1.12	0.75	Daboussi *et al.* (1992)
*Punt (Neurospora crassa)*	1.32	0.56	Magolin *et al.* (1998)

Standard index values for RIP are (TpA/ApT) > 0.89 and (CpA+TpG)/(ApC+GpT) < 1.03, (www.sourceforge.net/protects/ripcal).

The presence of a RIP silencing mechanism in the genome of phytopathogenic fungi may have a significant impact on the evolution of these organisms. For instance, in C. *heterostrophus* the mutation site is located in the transposons and in regions near the TEs (Ohm *et al.*, 2012). This mechanism may accelerate the rate of evolution in this genus, depending on the number of effector genes that are located close to TE-rich regions (Grandaubert *et al.*, 2014). Gene duplication is important for the evolution of a species and the presence of a RIP mechanism may have a significant impact on the evolution of several fungi. For example, the presence of a RIP mechanism is associated with the absence or paucity of duplicated genes in the *N. crassa* genome. In addition to creating one or more copies of a functional gene, a RIP silencing mechanism may also generate new alleles. Indeed, this mechanism is regarded as essential for the emergence of genes with new functions (Galagan and Selker, 2004).

In conclusion, TEs may play an important role in organizing the *S. sclerotiorum* genome and can potentially increase the adaptation of this species to different environments and hosts. Such adaptation makes control of the disease more difficult. Furthermore, the abundance of *copia* solo-LTR and TRIMs identified in *S. sclerotiorum* should facilitate the use of these sequences as molecular markers in future investigations of genetic variability in this fungus.

## Supplementary Material

The following online material is available for this article:

Figure S1 - Representative sequences of the elements *Copia-LTR_SS* and *Gypsy-LTR_SS*.

This material is available as part of the online article from http://www.scielo.br/gmb
