# Supplementary material for: Evidence of ectopic recombination and a repeat-induced point (RIP) mutation in the genome of Sclerotinia sclerotiorum, the agent responsible for white mold
Source: Genet Mol Biol. 2016 Jul 7;39(3):426–30. doi: 10.1590/1678-4685-GMB-2015-0241 (PMC5004834; doi:10.1590/1678-4685-GMB-2015-0241)
Supplement: Supplementary file 1 [file 1415-4757-gmb-1678-4685-GMB-2015-0241-Suppl01.pdf]

> *Copia-LTR_SS*

TGTTGACCTCCTGGGATCTTGGCAGCATATGCATATATAGTACATATGCATATAAGGAGCAAACAAAAC
TCAACTTTTAATTCATTCTTTCCTTTTTGGTATACTGTTTGTTTGCTTTCAATATTTTGATTTCAAAGCACTT
TAATTCCGAAGGCTGAATACCCAACCTATTCCTTCATTTTTTTGAATATATACTCGCAGCTTAACTGCTTA
GCTAGAGAATCAATCAATTCAATCTGATACTCTTTCTAGTCTCAAGAAACCCAACAGTTATGAGCCCGGT
GCTGACTTTCAATTAAACATCCTGCACAATACTTGATTCACATATACCGAAACTCTACGATACCTTCAAG
TTTCACTCTTTTAAGAATTACCTTACGTAGTGTGGTTTCTATAAAACCCAAACCTCGATAATTCTGACTTT
ATTTCTTCAATATGACTACAGGCGATAATTCAACTAAAATCATCCTTTCCTCACCAGAGGATTGGAATAG
TTGGATTCGAAATGTTCAGCGTATTGCAGAATCCGGCGGCATCAAATGCTGGGTATACCTTGATCCCAC
TACAACAGTTGTACCTATACCAGAGTTGCTAGATGAACCAGAGCTACCCACAGTTGATGAAAATGCTTC
TGAGGCTGTTATTGATAGGGCTTTAAAGCAATATCAGATGCAACTAGAGAACTATCGTATCAAGAATAC
CAAATACGAGAAAATCAACAAGGCCATTTCAGAGGTTCTCAAGTACATACTTTCATCAATCAACCGCAA
TTGCGAAAAGTTTATTAGTACCACCAGTATCACAGCACCTCAAGTGCTTGCTGATTTACGCAAAAGGCT
AGCACCAACTGATCGTGCGAGAGAGCTTACCGTCGTAGCACGATACAATTGTGTAAAGCAACCCTACA
AATCCAATCAGGATATTTATGAATGGTTGAACGAATGGGAACTCATCTACACTGAGGCTAAAACTCTCA
ATCTGCCAGACGTCGATCGAAATCGAGCTGCGTTCGACTTTTCTACAGCCCTTATGAATCACAGCTCCTC
TTGGGCAACTTCAACTCTGTTGGAGATCACCAAAAAGCTCAAGAATCAGCCTGAAGAAACGATTGATGT
GTACGATTTAATCGAAGACTTTCGAAACTACACTCGACTCACACAGGCATCAACTACTTCAACCTCTTCT
ACGTCATTTGCAACCTATAAGGATGGGAATAAAGATAAAAAGGAAGGAGAGAGAACCTGTCTATGTG
GCAAGAAACATCTATATGAAAAATGCTACTATCTATATTCGGATAATCGACCAAGGAACTGGAAACCAA
TCGATGCCGATATTGAATACTTCAAGAAGTTCTGGGGAAGGCAATCAACAAAACGAAGGAACTGGTTT
GTTCGTAGACATGAAGGAACTAAAGAACTTCTCAAGCTAGCAAATCTCTCTTGGGATCCAGCATGGAA
GGAAACATTGACTAATCCTTCAGAATCTGGAAAAGAGAAAGATAAGGATCTTGGGGCTTTTCTCACTAA
ACAAAGTCCTACCTACAACAATGCTAGCATCCTTCACAAATGGCTAATTGATAGCGGTTCCGATACCCAT
GTCACCAACTCTCTACACAACTTTGTTCTTGAAGAGTTTGGAAAGCCATCCGATATGATAAGCGCTGGA
AGGGACCGATTCCAAATTGAAGCATTCGGTACTATCACTATGTTGATCAATACACCAACTGGGCCAGGG
CAAATAACCTTAAAAAGAGTGGCGTACATCCCAGGGTTTCTTACAAACCTAGTCTCTCTAGCTTTACTCT
CAAAGAAAGGTGTACATTGGAACACTGAACACCCAGATGTCCTTACAAAAGAAGGCATTGCATTTATTA
CTCTGAAGCAAACAGGAGACCATTGGACCCTCGATCCTATTATCCTTTCTCAGCATGCTTCGTTTGCCAC
CAAAAACCACACCAAATCAATATGCCGAAAGGTTACAACAACAGAGTTACATGATCTTCTAGGCCATCC
TTCTCTCGAAGCCCTTCAGCACCTAGAATCAGCAACTACTGATATCATTATCGACAAAACGATTTCGACA
CCTTCCTTCTCTACCTGCAAAGTTTGTAATCTCTCGAAGGCTCAACAGATTATATCTCGTAATCCAAGGA
AGGAGATGCCAGAGAACGGCCATGTCGGAGATCGATTGGCATGGGATCTGTTCGAGATGGATGAGGC
TTATAATAATACCCGATATATCAGCCACTTCTGGTGTACGACACACAAAATACAATGGGTGTATGTTCTA
ACTAGGAAAACAGAGATGATCGATATTATGGATACTCAATTCTCTCTGATACAAACACAATTCGGCTCA
AAGGTCCGATTCATACGCCTCGACGGTGAAACTACGTTAGGGGGCGACTTCAAGAGGATCACAGACAA
ACGTGGCATCATTGTCGAACGCACTCCACCAGATACACCAGCACAGAATGGCGCTGCAGAGAATGCTG
GTAAAATGCTCATGGTCAGAGCAAGAGCCCTCCTCCTTTCAGCAAACCTACCACAAAACCTTTGGCCAG
AAGCAATTAAAACAGCCGGTTATCTTGAAAACCGTACTCCAAAAAGAGCTCTTCAATGGAAAACACCAT
TTGAAAGTGCTACTGGCAATAAACCTTCGCTAGCACATCTACATCCATATGGGTGCTTGGCATATGTGCT
CAACAAGCAGATTCCAAGACGTGCGAAGATGCAACCTCGTGCAAAACTGGGCTATCTTGTAGGGTATG
ATTCAACGAATATCTTCAGGATCTGGCTCCCTTCCATAAATCGCGTCATTCGAAGTAGGGATGTCAAATT
TGATGAAACCCGGTATTATCGACCAGAAGATATAGATATTGGCTTTCTTATTAAGGATCAACTTCGGGA
TACTGCAGCTATTCACGAAAAGAGGGCAGAAGAGATAGAAATATCGAATCTGTTGAGTGAAGAATCAG

ATGAGAGTATTGGTGATATAATTGTAGTTGAAGGGCCATGGAACTTACAACACAATCGCTTAAACGAG
CGACCAACGAATGAAAGCTCTTCAAACAAGACGGATGATGGTATTCCAACCAATGAGGGGGTTCCTCA
AACTAACCCTCAATTGCCAACGCCTAGAGCAACCTTATCACCAGAAGCACTTACAAGACAATCTACTGG
AGATGTTCCTATACCATCATTACCGGAGTCAGAACTCTCATCTTCCCCATCATCATCGTTAAGTCAACAT
GATATCATCACTACAACATCATCTCATAATACAGCACCTTTGGCAGAGCAAATACGTGCCGATATCGAC
GTATCCCATATAATTCAGGGTTCTAGACGTTCACAAAGGAAAGAACATCATGCTGCAGCACTTTCATCA
CTTCCTCAACTATCCAGCTACTTTGCAGCATTTGCTATCGGAAGGTCTAAATTACGAACTCCTAGATTAC
ATCAAAATGATCTACCACCTCCACCTCGATTTTATCATGAGCTAGTACAGCATCCATATGCTAGAGAATT
CCAAAAAGCATGTGAGGATGAGATCAACGCTCTGAAAGAACGTAAAACCTTCGAATACATCGATATAT
CTACAAGAGGACAAAAGCCTTTACCATTACTCTGGGTTTTCACTTATAAATTCGATCAAGATGGTTATCT
CATCAAGTTCAAAGCAAGAATCTGTGCCAGAGGAGATCTTCAATCAACCGAAGAAGAGACATACGCTG
CAACGCTAACTTCACAAATTTTTAGAGCTATGATGGCTATCGCTGCAGCTTTCGATCTTGAGATTCGACA
ATTTGACGTTTCCAATGCTTTTTTACATGCAACATTAAGGCAACCAATTATTTGCAAGTGCCCTGATGGC
TTCGAAGTCCCAGGGAAAGTGATTTCCGTAAACCAAGCACTTTATGGCTTCCGAGAGTCTCCAGCATAC
TGGTATGAGGATCTGACCTACACGTTAACCGACTTTGGTCTGGATCCAGTTCCAGGAGCACCCTGTGTT
TTCACCAATAGCTGGCTTACGGTTCTCTTCTATGTCGACGATATTATCGCAATTTGCAAGAAAGCTGATT
TGCTACGATTAACCTTATTCGAAAACACCTTAAAATCAGCATACACCATCAAATCACTTGGAGATATACA
GTTCTTCTTAGGGATTAGGGTACTACGAGATCGACAAAATCATCGTCTGTGGCTTTGCCAAGATAACTA
CATTTCAAAAATATCAACACGCTTCAATATCAAACCTAATCCTCAAGCCTGTTCTCCATTGCCAACAACA
GCACTTACAAAGAATCCAGCAATTGCAACTCCGCAACAGGTTTTCGGCTACCAGCAGCGAGTTGGCAG
CATTAACTACGCTGCTGTTGTGACTCGGGCTGATATTGCAAAAGCATCTTCAATACTATCAGAATTTCTT
CGTAATCCATCCGCTACTCATATCAATGCAGCAGTACATACATTGCAATACCTTGAGAATACCAAATTCT
ATGCTATCATGTTCGATGGGAAGAAAGCAACAACTACCAGGAACTTTCAAATCTGGAGTGACGCCTCCC
ATGCTGACGATATCGATACAAGAGTTTCTTCAATGGGATATTGCCTCATACTCTTTGGAGGTTTGATCCA
CTTCAAAAGCACTAAGCTGAAGTCGGTGACTACTTCCAGCACACACTCTGAAATACTGGCTCTTTCCGTT
ACTGCTCGAGAGCTTATGTGGTGGATACGCTTCTTTAAGAATGTTGGTTTAGATCTCGAAGAAAAGGCT
TCAATTTTCTGTGATAATCGCCAAACCATTCGACTTCTTCAGCATAGTACTCCTAGACTTGTTACAAAACT
CCGGCATGTTGATATACACTCCTGTTGGCTTCGCCAGGAAGTGCAAGCAGGAAGGCTACAAGTCGAAT
GGGTTCCAACTACTGAAATGGTTGCAGATGGTTTCACTAAACCTCTACCACCACAGAAACACACTCATTT
TATCAATCTTTTGGGAATGGTTGACATTCGGTCTCAGGTTCAGGAATAACCTCATTATTAACTAACTCGG
TAATCCCTGAGGGGGCGTGTTGACCTCCTGGGATCTTGGCAGCATATGCATATATAGTACATATGCATA
TAAGGAGCAAACAAAACTCAACTTTTAATTCATTCTTTCCTTTTTGGTATACTGTTTGTTTGCTTTCAATAT
TTTGATTTCAAAGCACTTTAATTCCGAAGGCTGAATACCCAACCTATTCCTTCATTTTTTTGAATATATGC
TCGCAGCTTAACTGCTTAGCTAGAGAATCAATCAATTCAATCTGATACTCTTTCTAGTCTCAAGAAACCC
AACA

> *Gypsy-LTR_SS*

TGTTACGAGGTTTGCCGTCTAGATCACGTGGAAGTGCTCCAGGCTCTCGAGGTGGTTTATAACTAGGCG
TCAAGCAGGGACTTAAGTAATGACAATAATGATATGCAATTCGTAACTAATAGTAGAGTATACTAAATG
ATTGATCTTGAGCACGAAGCTCTACACAAAGGAAACCATCCTGATTATATAGACCTTCGTCCTCCATTGG
ATCTCTTGGATCCTGAGAGTTTGTGGACCTTCGATTCGTATGGACCTACGATTTGTATCCCTGAATATGC
AGGGTCCATCACGTGACTTGGATCAGACTATATGTCTGTGCTTGCTGAAGGTGGGGAGACTCTACGAA
CCGATTACCATACAAAATTGTATGCGAATCGGTTTGCTTTTCCACGGGGTCTAAGCTCATATGGACAGG
TTGACTGTTACTACGTAACACTATCCCCTTCTTAGCCTAGTGCTTGTCCCAAGCTCTTATAGTCACGTTTC
CTGCTGTGTTATGATTTTTCGGAAGATAACTTTTCTTCTTTTCTTCCATCATGCCACCTGTACGATCAAAG

AAATATAAAGAACGAGTCGAACTTGCTTTAATAATTGAATCTGAAGGGTTCGAGATGCCTCCATGTACT
TATTGTGAAAAACATTCACGTCGTTGTATTGTTGCTAAGGAAAATTCCAATCGTTGTAGTGAATGCGCTC
GTCGGGGCCAGCGATGTGATATATCGGGTCCTTCTTCCGGAGATATGACGAGCATTATTCAGGAGCAG
GAGCGTTTGGATCGCGAGCGCGAAGAGACTTTGTCGAAATTGCTTCGTCTAGATAAGCAACAACGCTT
CCTTCGTGGGCGGGCTTCTGAAATGATTCGTCGTGGTTTAAAAACAATGGATGAGTTGGATGATGCAG
AGGAGAAGGAAAGATTGGGTAAGGAGCGTATAGGGAAGGAGCAGTCAATTTCTGGGTTGTCAATGCC
TGCTGAAGCTAGTTCTGATGTGCTGGATAACTTCAATCCTTCTTCTTTCCTGAGTCCTTCGGACCCCTTTT
CCTTTCAGAATTCTTCGGGTCCTTTTTGGGGAGATCCGGATAGCGTTGGTGAAATGCCTCCAACTTCTCA
GGGCAGCTGAGATCTTTCTTGAGCTCCCAAGTGTTTTCTGAATGTGGGTAATCCAACCATTTGATCAGA
TATTTGATCTTGCCTCTGACGTATTTACAATCTAGTATGGTTTCAACGTCGTATATGGCGTCTGGGTTGA
CTGGTTCGATTTCCGTTATTGGCGCATCGGGCGCTCCTGGTGGTGCTGGTTCAAGCAAGGATATATGGA
ATACTGGGTGAATATTCATGGTGTGTGGTAGTTTTAGTCTATAGTTAACTGGTCCAATTACCTTGTCGAT
TTTAAATGGTCCTAGTTTCTTGTGATCTAGTTTATTGCATGGGTCGTTTTGTCTGTATGTTTCTTCGAATG
AGATAAACTTTATCCCCCTCTTTAAGCGTAGGTCCTATACTATATGATTCGTTGTAGTACGACGCTGTTCT
TAAAGCGATGAACTGTAAATCCAAGGAAAGTTGTTCTTGTAGTTCCTTGATTTCTGTGATATCTTTTATG
GCTTCGTCTGCATTCGCATCTTGTAGCAATGGTGATTTATATGCTAGTGGATTGAACCCGAAGTTAGCAT
AAGCTGGCGTCACTTTTGTTGTTTCCGTTTCCGATGTATTATATGCGAATTGTGCCAAGGGTAATAGTTT
AACCCAATTGTCTTGTTTGTAATTGATGTAGCAGCGAAGATAGGCTTCCATCGTTTGGTTTAATCGTTCC
GTTTGGCCATCTGTTTGTGGGTGAAAGGCTGTCGAGAGCTTTCTCTTGATTCCAAGCAGTGCCATAAGG
GTAGACCAAAATTTCGATGTGAATAGCTTGTCCCTATCTGAGATGATTTCATCTGGTACACCGTGTATGC
TAACTATATTCCGCATGAATATATAAGCAAGGTCATCTGCCGTCCAAGTTTCACAGAATGGTATCATATG
AGCAAACTTGGTTAGTCTGTCCGTCACCACCAAGATGGAGTCGTACTCCTTCCGGGTGACTGGGTCTTT
GGATAATGGTAGTTTTACTACAAAATCCCAAGCAATGGACTTCCATGGTTGAGTAGGTAGTTCGGGGG
TCTTGAGCTGGCCATAAGGTGCATGCCGGGCGGCTTTATTTCTTATGCAGGTGTCGCAGTTCCCAACTA
CGTTCTCAACTATAGTCCTCATCTTTGGAAAGTAGACTTCTCTCCTTATTCTGGCAAAGGTTCTTGCAATA
CCTTGATGCCCGTGAGCTGGAAGCTCATGTTGTTCCGTGACGTAATCCTTAGCTAGACTTGAGGGTATG
TAAAGCTTGCCATGAAAATGGATGAATCCATTTTCGATGGTGAATCCTCTTTCTGGATTGTCTGGTATAG
CTAGGATTCTCTTGGCTGTAGTGTCTGTTGAGTATTCTGTCTTTATGATATCTTTGATAGTATCATAATTT
ACTCCTGATGTGGCGGCAAGTTGTGGTTTGTTGTGAACTAAAGCGTCGCCATCCTGCTTTAGTATTGCG
CGTGATTCGTGTGTTTTGTTTTGTAAGTATTCTGGTTTTCGGCTGAGAGCATCTGCTCTAGCATTTTCGTT
TCCTTTGACATATGAAATTCTGAAGTTGTAGGCGGCCATGGTTTCCGACCATCGGACCTGCCGTCTGTTT
AATTCCTTCGTTGTGGTGAAGTAAACCAAGTTCTTGTGGTCTGTATACACCTGCACCGTGTATTTTGATC
CTTCCAAGTACACTCTCCATTCCCTAAAAGCATCTACTATCGCTAGTAATTCCTTGTCGTATATCTCATAG
TTTAGTTCGGCTGGCGATAGCTTGCGTGAATGGTAAGCGACTGGTTGCTATTTCTCATTCGAGCCGGGT
TGGCTCAACACTGCTCCTATTGCGTAGTCTGAGGAGTCTGTTTCCACGATTATTGGTAATTCTGGGTCAA
ATGTTCTGAGGACTGGTTCTCCTGCGAATGCTTCGTTGAGGCGTTCGAATGCTTCTTGTGCTGTTTCTGT
CCATCGGAATTCTCCTTGACTCTTCTTGGTGATCTCGGTTATTGGAGCGGCTATCTTTGAATAGTTCTTG
ATGAATCGTCGGTAGAAGTTGGCGAACCCCAGGAAGGACTGTACTTCCTTCACGCTGGTTGGGGGTTC
CCATTCCATAACTGCTTTTACCTTGTTAGAACTCATCTTGAGACTTCCGGAGAATATGGTATAGCCTAGG
AATTCCGTTTCTGTGACGTGAAATTCACACTTGCTAGGTTTGCATTGAAGATCTGCCTCCTCCAGTTGCT
GCAGAACCTTATGAACGTCTTTAATGTGTTGTTCTTCCGTTTCCGAGAATACCAAAATGTCATCTAGATA
GCATATACAGCATATATCTAGGTACTTCCTTAATACATTGTTGACCAAGCGCATACATGTTGCTGGTGCG
TTTGTCAATCCGAATGGCATAACCTGATACTCGTATAATCCGTATCGAGTTCTGAATGCTGTTTTCCATTC
TTCACCTTCCTTCATTCTGATGAGGTGAAATCCGTTTCGTAGATCAAACTTGGAGAAAATTTTAGCTTTTC
CCAATCGATCCCTCAGTTCCGTAGCTAATGGAAGTGGGTACCGATCTTTGATCGTTATATCATTGAGCTT

GCGATAGTCTACGCACAGTCTCCTTGTACCATCCTTCTTTGGTACAAATAGCACAGGGCTAGCTGCCTGT
GAGGTGGATTCTCTGATCCATCCTTTTTCTAATGCGTCGGCAATGTAATCCCTAAGCGTCCTTAGGTCAG
AGGCTGACATTGGATAGATTGGTCCAAATGGAGGCGTCTTGCCTTCCTTCAATACTATCTCGTGATCCCA
TGGCTTGTGTTCTGGTAAAGCTTCATGGCCTTTTCTTTCTTCGAATAGTTTCTTGTACTTCTGGTACTGTA
CCGGTATTGGCGTCTCGTCCTGTTTCTCTACAGGGGTGGTTTCAAGCAACTTCTTCTGCTTATTCTTCATT
TGTACGGGAGGTTCACCTTCCTTCGCCGTTAATGGAGGACCCTGGACTGGGCGTCCTACGTCAGTGGT
GCTGTTCGCCTTCAGTTCTCCACCGCGTGTGTTCAGCCTCGCAGGCTCTAGCGCATCTCGTTGATCGCTT
CTCCTCGGAAATGATATAGTTTCCGATGACCAGTCAATCTGGGGGTTGTGTGCTTTCAACCATGGCATC
CCCAGAATGATCTGTTGATTCCCTAGTGGAACAACATCGAACTGGATAACCTCCTTGTGCTGGGTTATA
CTCATCTGCAAAGGGCGAGTCTCCTGCGAAATCTTTCCGTCCAATCCGGCTAATTGTCCATCAACTAGCT
GCAATTGATATGGGGTTCGTTTCGCCTGGAGCTGTACTCCCAAATGCTTCACAGTTCTTGGTGCAATAA
AATTTCCCGTGGCCCCTGAGTCAATCATGGCCCGGATTACTCGTCCATTTATCTTCGCATTGCAGTAAAT
CTGTCCTGCCTGACCTGCGATATTTAATTGTCCTTGACCCAAAACCTTGTTGTGGTCAATTTGGTCGATTT
GGTCAATTTGGTCGATTTGGTCAATTTGGTCGATTTGGTCAATTTGGTCAATTTGGTCAATTTTGTGTAA
CTGACCATTTAAATGATCATGTGACGATAATTTTTTCGACCTTCCCTTCTTGTAGGGTCCTTTTGCTTTTCT
TTGGGGAAACCATCCTGTGGCTTCCTTATCTGATAGGTGAATTCTACAAGATTCATCGTAGCAAGCTATC
CAGCTCATCGTCGCGTGTTCTACCGACGGTATTTCTTTCGGATCTTCTTTGATAAGGGGAGGTGAAGGG
GTTCGTAGACGCTCCAACTCATATCGGTCTATGGCGTTCAACCCTTCCTGGTCTCGTGGCATTGCGTCAA
TTTCCTTTTGACGTTCTTCACGTTCGTAACTATCGTCTTCGATTGGATATGAAGCGCATAGTTGGGGTAC
AAAGGTAGCATTGAGTTGACCTTTGCCGAACTTCTTGTGATAACCTCCTCCTGATGATCTCTTGCTTCGA
CAATCCCTGGCCCGATGGCCTGGTTTGCCACATTCGAAGCATAGCTTGTCCTTGCGTCTTCGTTCTACCT
CTTCCTTAGGTAGTTTGTTCTTAAACGTTGCATCCAATTGCATTGGTTTTCCGTAAGATTGTCTGTTTTCT
CGAGGTCTTCCTTCATTCTTGTGGTACTTACCACGATCTCGGTTTCCATGGACGTTGGAAGATCGGCGTT
CCTTCTGATACTCGTAGAGCATCTCATCCGCCTTAATAGATTCCTCGATTAACTTGTTTAGGTCTTCCCAA
GTGCCATTGCGATCCAGTTCTAGCCTGACCTCAGGCTTGAGCCCTTTCCGGTAATGGCTCAACGTCGCG
TTCTCTTCCCATCCAGTGGTACCTATATACCTCCTGAATTCCACAGCGTACTTGACTGCTGACCCTGTTTG
CTTGAGGCCATAGATCTTGGACTCAGCGTCACGTAATTCATTGATGTTCCCGAATACTCGTCGTATCTCT
CTCTTAAACCCTTCGAATCTCCCAAAGATGGCTTGAGTTGGGTACATTCGCTTCTTAGGCTCGCCCAAGT
TGGTGAAGTAATCCTTGAGGTAAGGTTGAATCCACTTTGCGGCTTCTCCTCTGAGGTAGGAGGCAGCCC
AGACGCTTTTGTCGGCGTCATGGTTGAACTTATCCTGATTGAATTGGAAGTAGATCTCAAGTTGGAGTA
AGAAGGTTTCTAGCTCTTGTCGATTGCCTGTGAATCGGTCTGGTAGGGCGACTTTAAGAGTCTCGGTGA
CGGTAATCTCCTTGGGGTCTTCGATACCTCCCATAGCGATGTCTCCGTCCTTGTCGGTACTTTCGGGAGT
GGCCTGGTTGGTCGTAGCGGGGTTGCTAGGCTGTGGTGTCGTGGAAGACATGATGTCGTTACTTATCG
AGTCGGTACTTGAAGCAGTAATGGTGCTCAAAGTGTTACGAGGTTTGCCGTCTAGATCACGTGGAAGT
GCTCCAGGCTCTCGAGGTGGTTTATAACTAGGCGTCAAGCAGGGACTTAAGTAATGACAATAATGATA
TGCAATTCGTAACTAATAGTAGAGTATACTAAATGATTGATCTTGAGCACGAAGCTCTACACAAAGGAA
ACCATCCTGATTATATAGACCTTCGTCCTCCATTGGATCTCTTGGATCCTGAGAGTTTGTGGACCTACGA
TTCGTATGGACCTACGATTCGTATCCCTGAATATGCAGGGTCCATCACGTGACTTGGATCAGACTATAT
GTCTGTGCTTGCTGAAGGTGGGGAGACTCTACGAACCGATTACCATACAAAATTGTATGCGAATCGGTT
TGCTTTTCCACGGGGTCTAAGCTCATATGGACAGGTTGACTGTTACTACGTAACA

Figure S1. Representative sequences of the elements *Copia-LTR_SS* and *Gypsy-LTR_SS*.
